# Supplementary figures and images for: Dual human lung models reveal compartment-specific activity of anti-tuberculosis drugs and host-directed therapies
Source: Microbiol Spectr. 2026 May 11;14(6):e03729-25. doi: 10.1128/spectrum.03729-25 (PMC13227965; doi:10.1128/spectrum.03729-25)

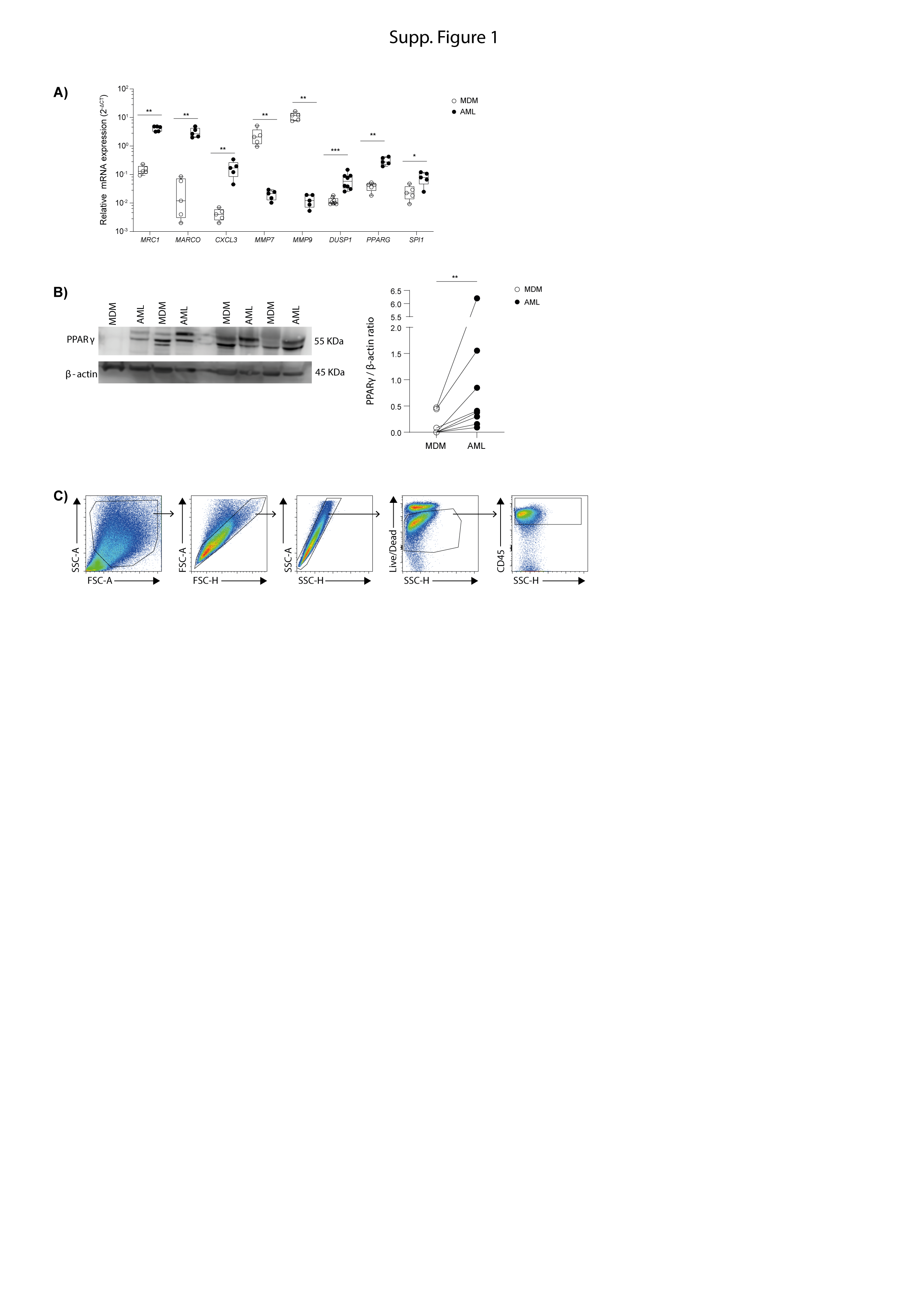

Supplement: Fig. S1 — Molecular characterization of AML and MDM and flow cytometry gating strategy for phenotypic analysis. [file spectrum.03729-25-s0001.tif]

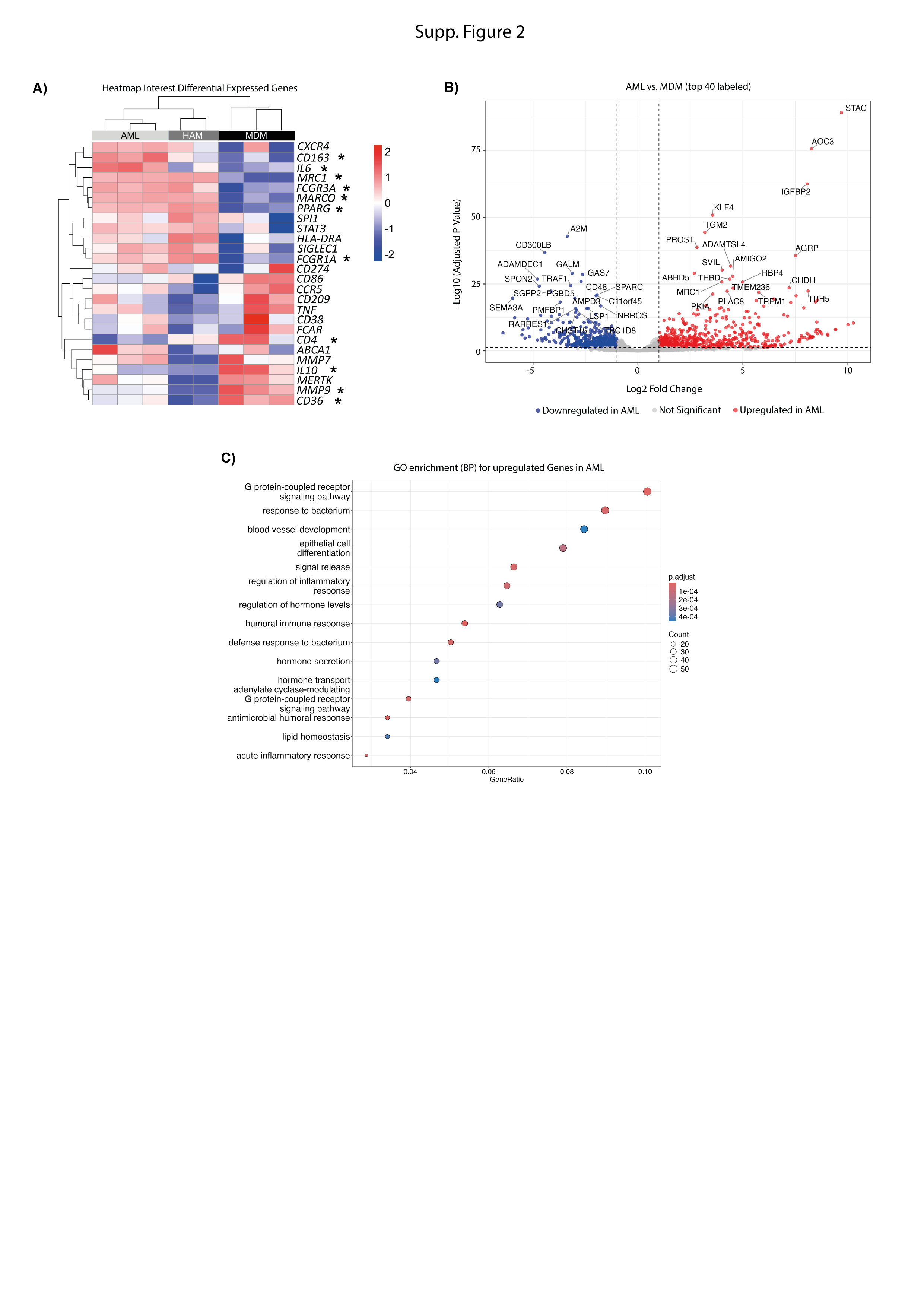

Supplement: Fig. S2 — Differential gene expression analysis of AML versus MDM. [file spectrum.03729-25-s0002.tif]

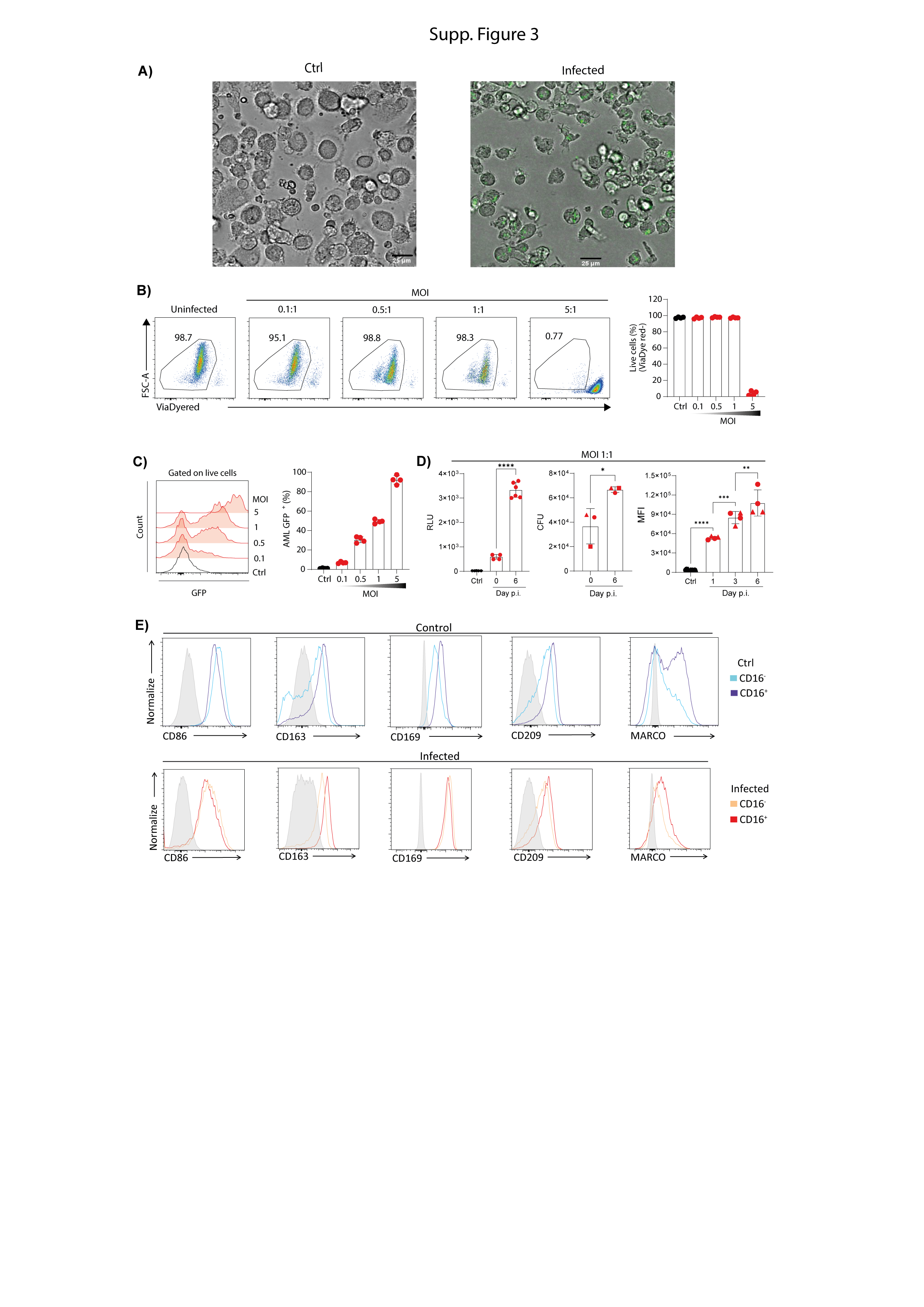

Supplement: Fig. S3 — Optimization of infection conditions and phenotypic profiling of AML cells upon Mtb infection. [file spectrum.03729-25-s0003.tif]

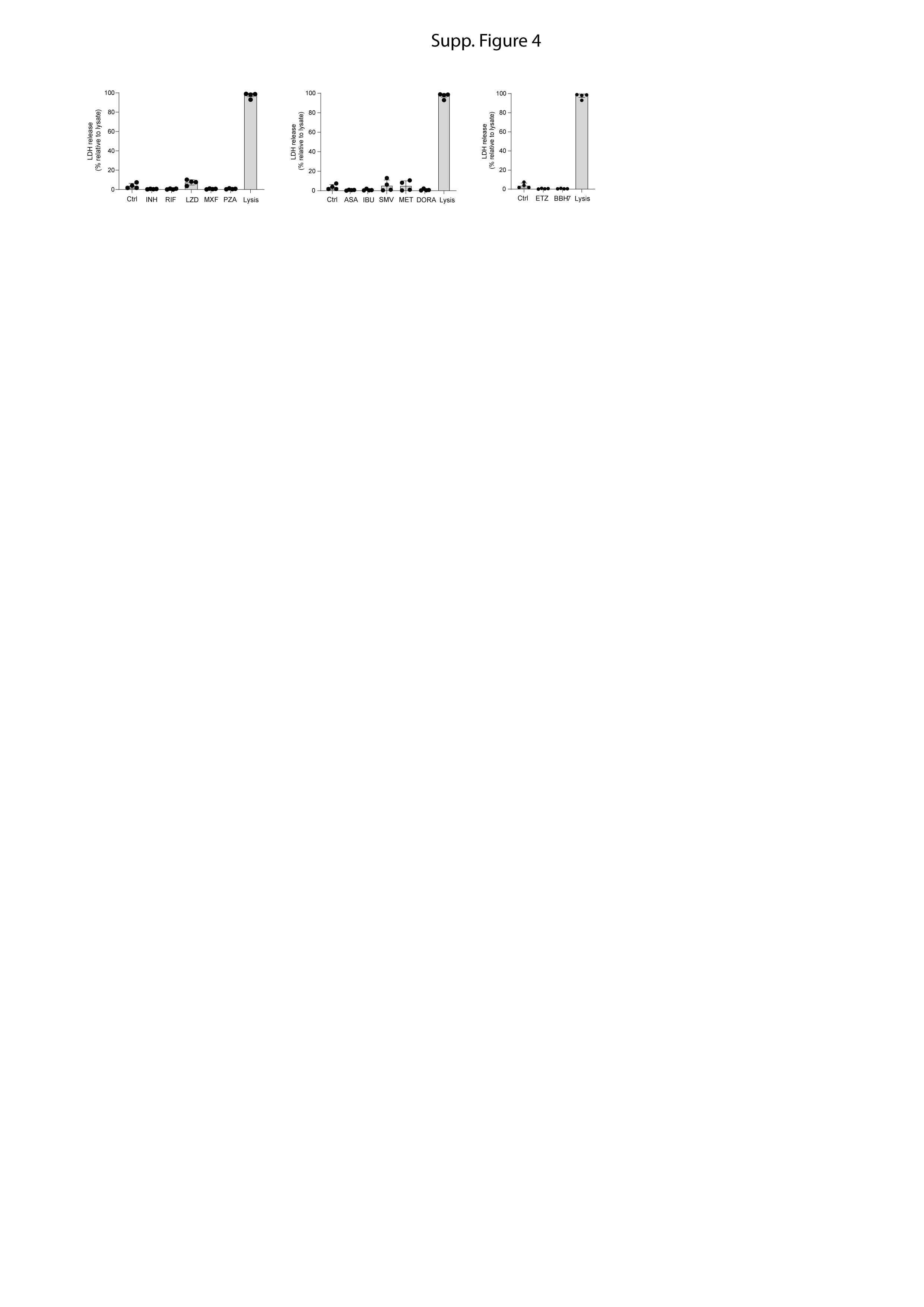

Supplement: Fig. S4 — Evaluation of toxicity induced by anti-TB agents in AML cells. [file spectrum.03729-25-s0004.tif]
