# Supplementary material for: Dual human lung models reveal compartment-specific activity of anti-tuberculosis drugs and host-directed therapies
Source: Microbiol Spectr. 2026 May 11;14(6):e03729-25. doi: 10.1128/spectrum.03729-25 (PMC13227965; doi:10.1128/spectrum.03729-25)
Supplement: Supplemental material — Supplemental figure legends; Tables S1 to S4. [file spectrum.03729-25-s0005.docx]

**SUPPLEMENTARY MATERIAL**

1. **SUPPLEMENTAL FIGURE LEGENDS**

**Figure S1. Molecular characterization of AML and MDM and flow cytometry gating strategy for phenotypic analysis. A)** Relative gene expression in AML and MDM cells assessed by RT-qPCR. **B)** Immunoblot analysis of PPARγ expression in AML and MDM cells from four independent donors. Quantification of protein levels is shown. **C)** Flow cytometry gating strategy used for the analysis of hAMs. In the graphs, each dot represents an individual donor. Significant differences were determined using a paired, non-parametric Wilcoxon test. p < 0.05 (**), p < 0.01 (***), p < 0.001 (***).

**Figure S2. Differential gene expression analysis of AML versus MDM. A)** Heatmap of the principal representative genes. The heatmap shows differences in expression across the samples. Rows correspond to the different genes and columns to the different samples of AML and MDM cells. Red color is indicative of up-regulated genes, and blue is down-regulated; AdjPvalue < 0.05 is shown with *. **B)** Volcano plot shows the top of principal 40 DEG (FC +/- 1.5 and AdjPvalue < 0.05).  The blue dots denote down-regulated genes, and the red dots denote up-regulated genes in AML. Grey dots are out of range. **C)** Gene Ontology (GO) enrichment analysis of biological processes (BP) for up-regulated genes with the principal deregulated genes between AML vs MDM cells. The dot size indicates the number of genes, and the colors correspond to the Adjusted P-Value.

**Figure S3.  Optimization of infection conditions and phenotypic profiling of AML cells upon Mtb infection. A)** Uninfected and H37Rv-GFP-infected AML were visualized using epifluorescence and light microscopy. Bacteria are shown in green. Scale bars: 25 μm. **(B-C)** Uninfected and H37Rv-GFP-infected AML (MOI of 5:1, 1:1, 0.5:1, and 0.1:1) were analyzed by flow cytometry. The percentage of live cells (B) and GFP+ cells among live cells (C) is shown. **D)** Measurement of bacteria proliferation in infected AML (MOI 1:1) after 0-, 1-, 3-, or 6-days post-infection (p.i.) by using H37Rv::lux luminescent bacteria (left), colony-forming unit (CFU, center), or Median Fluorescence Intensity (MFI, right) of H37Rv-GFP strain. **E)** Median Fluorescence Intensity (MFI) of CD86, CD163, CD169, CD209, and MARCO of CD16^+^CD14^+^ and CD16^-^CD14^+^ subsets in uninfected and H37Rv-infected AML cells. Significant differences were determined using an unpaired, non-parametric Mann–Whitney test. p < 0.05 (*), *p < 0.01* (**), p < 0.001 (***).

**Figure S4. Evaluation of toxicity induced by Anti-TB Agents in AML cells.**

Lactate dehydrogenase (LDH) release was measured as a marker of cytotoxicity. Mtb-infected AMLs (MOI 1:1) were treated with different compounds and incubated for 6 days.

**Supplementary Movie 1:** Ciliary beating of airway ALI culture visualized using light microscopy (EVOS M7000 - Invitrogen) with a 60× objective. Real-time recording (live) showing coordinated cilia movement.

**2.**  **SUPPLEMENTARY TABLES**

**Table S1. List of commercial drugs used.**

| **Name of the molecule** | **Mechanism relevant to TB control** | **Compound class** |  |
| --- | --- | --- | --- |
|  |  |  |  |
| Isoniazid (INH) | Inhibits mycolic-acid synthesis (75, 76) | Standard-of-Care  (SoC) |  |
| Moxifloxacin (MXF) | A fourth-generation fluoroquinolone antibiotic that inhibits DNA replication in Mtb by diminishing DNA topoisomerase II and IV activity (77, 78) |  |  |
| Pyrazinamide (PZA) | A first-line agent whose activity and mechanism are still under investigation, with current evidence suggesting roles in disrupting membrane transport and proton motive force (59, 79) as well as other targets (80) |  |  |
| Rifampicin (RIF) | A first-line ansamycins antibiotic that specifically binds to the β subunit of bacterial DNA-dependent RNA polymerase, thereby inhibiting transcription (81, 82) |  |  |
| Linezolid (LZD) | An oxazolidinone antibiotic that inhibits bacterial protein synthesis by binding to the 50S ribosomal subunit (83, 84) |  |  |
| Ibuprofen (IBU) | A non-steroidal anti-inflammatory drug (NSAID) that inhibits COX-1/2 and reduces PGE₂ synthesis, enhancing phagocytosis, nitric oxide (NO) production, and NADPH oxidase activity (44, 85) | Host-Directed Therapies (HDT) |  |
| Aspirin (ASA) | A NSAID with antiplatelet, anti-inflammatory, and analgesic properties that inhibits COX-1/2 and reduces PGE₂ synthesis; additionally, it promotes the formation of aspirin-triggered lipoxins that help resolve inflammation (85, 86) |  |  |
| Doramapimod (DORA) | Selective inhibitor of p38 MAPK, reducing pro-inflammatory cytokine production (43, 87) |  |  |
| Simvastatin (SMV) | Enhances phagosome maturation and promotes apoptosis and autophagy in Mtb-infected cells (88–90) |  |  |
| Metformin (MET) | Induces reactive oxygen species (ROS) production and increases phagosome–lysosome fusion (91) |  |  |
| Ethoxzolamide (ETZ) | A carbonic anhydrase inhibitor that has been repurposed for TB research by targeting the PhoPR two-component regulatory system in Mtb (92) | Virulence-Targeting Therapies (VTT) |  |
| BBH7 | Inhibits the ESX-1 secretion system, impairing virulence factor export (70, 93) |  |  |

**Table S2. List of SYBR Green human primers used for qPCR.**

| **Primers** | **Gene reference** | **Sequences - Forward (top), Reverse (bottom)** |
| --- | --- | --- |
| *MRC1* | NM_002438.4 | GCCTCGTTGTTTTGCGTCTT |
|  |  | GAGAACAGCACCCGGAATGA |
| *MARCO* | NM_006770.4 | AGGAGGACGAGCTCTTGAGT |
|  |  | TCAGAACTTGGACCACCAGC |
| *CXCL3* | NM_002090.3 | CCCAAACCGAAGTCATAGCCA |
|  |  | ACCCTGCAGGAAGTGTCAAT |
| *PPARG* | NM_138711.6 | GGTGACCAGAAGCCTGCATT |
|  |  | CACGGAGCTGATCCCAAAGT |
| *DUSP1* | NM_004417.4 | GTACTAGCGTCCCTGACAGC |
|  |  | CCCAGGTACAGAAAGGGCAG |
| *SPI1* | NM_001080547.2 | AAAATCAGGAACTTGTGCTGGC |
|  |  | GGGGAAACCCTTCCATTTTGC |
| *TNF* | NM_000594.4 | GAGGCCAAGCCCTGGTATG |
|  |  | CGGGCCGATTGATCTCAGC |
| *IL10* | NM_000572.3 | ACTTTAAGGGTTACCTGGGTTGC |
|  |  | TCACATGCGCCTTGATGTCTG |
| *MMP7* | NM_002423.5 | GTCTCTGGACGGCAGCTATG |
|  |  | TAGTCCTGAGCCTGTTCCCA |
| *MMP9* | NM_004994.3 | GGACAAGCTCTTCGGCTTCT |
|  |  | TCGCTGGTACAGGTCGAGTA |
| *IFNA* | NM_024013.3 | GTGAGGAAATACTTCCAAAGAATCAC |
|  |  | TCTCATGATTTCTGCTCTGACAA |
| *IFNB* | NM_002176.4 | AGCTGCAGCAGTTCCAGAAG |
|  |  | AGTCTCATTCCAGCCAGTGC |
| *MX1* | NM_001144925.2 | CGGAATCTTGACGAAGCCTG |
|  |  | CCTTTCCTTCCTCCAGCAGA |
| *MX2* | NM_002463.2 | CAGAGGCAGCAGACGATCAAC |
|  |  | TTGGTCAGGATACCGATGGTC |
| *ISG15* | NM_005101.4 | CGCAGATCACCCAGAAGATCG |
|  |  | TTCGTCGCATTTGTCCACCA |
| *SIGLEC1* | NM_023068.4 | ATGGGGTACGCCTCCAAAC |
|  |  | GTGCCTCATTGGGTGTGTTG |
| *YWHAZ* | NM_001135699.2 | CCTGCATGAAGTCTGTAACTGAG |
|  |  | GACCTACGGGCTCCTACAACA |

**Table S3. List of antibodies used for Western Blotting.**

| **Antibody (species)** | **Dilution** | **Supplier** | **Reference** |
| --- | --- | --- | --- |
| PPARγ (rabbit) | 1:1000 | Cell Signaling Technology | 2435 |
| β-actin (mouse) | 1:1000 | Merck | A1978 |
| Anti-rabbit (goat) | 1:5000 | Advansta | R-05072-500 |
| Anti-mouse (goat) | 1:5000 | Advansta | R-05071-500 |

**Table S4. List of antibodies used for spectral Flow Cytometry.**

| **Antibody** | **Fluorochrome** | **Clone** | **Supplier** | **Catalog #** | **Dilution** |
| --- | --- | --- | --- | --- | --- |
| ViaDye | Red | - | Cytek | R7-60008 | 1:4000 |
| CD206 | AF700 | 15-2 | Biolegend | 321132 | 1:400 |
| CD14 | CfluorB548 | 63D3 | Cytek | R7-20116 | 1:200 |
| CD169 | BV421 | 7-239 | Biolegend | 346018 | 1:100 |
| CD4 | BV650 | SK3 | Cytek | R7-20166 | 1:100 |
| CXCR4 | PE-Cy5 | 12G5 | Biolegend | 306507 | 1:50 |
| CCR5 | BV785 | J418F1 | Biolegend | 359131 | 1:50 |
| CD209 | APC fire750 | 9E9A8 | Biolegend | 330115 | 1:100 |
| CD86 | BV605 | IT2,2 | Biolegend | 305429 | 1:50 |
| PDL1 | PE-fire810 | 29E,2A3 | Biolegend | 329755 | 1:100 |
| CD64 | BV650 | 10,1 | Biolegend | 305053 | 1:50 |
| CD163 | PE-Dazzle594 | GHI/61 | Biolegend | 333623 | 1:100 |
| MertK | BV711 | 590H11G1E3 | Biolegend | 367619 | 1:200 |
| CD16 | CfluorV450 | 3G8 | Cytek | R7-20184 | 1:50 |
| MARCO | PE-Cy7 | PLK-1 | Thermofisher | 25-5447-42 | 1:20 |
| CD36 | PE | CB38(NL07) | Thermofisher | A15793 | 1:20 |
| ABCA1 | Dylight 650 | polyclonal | Thermofisher | PA5-22907 | 1:20 |
| CD45 | BV510 | HI30 | Biolegend | 304035 | 1:50 |
| CD38 | PE-Fire640 | S17015F | Biolegend | 397217 | 1:50 |

75. Winder FG, Collins P, Rooney SA. 1970. Effects of isoniazid on mycolic acid synthesis in Mycobacterium tuberculosis and on its cell envelope. Biochem J 117:27P. https://doi.org/10.1042/bj1170027pa

76. Timmins GS, Deretic V. 2006. Mechanisms of action of isoniazid. Mol Microbiol 62:1220–1227. https://doi.org/10.1111/j.1365-2958.2006.05467.x

77. Naidoo A, Naidoo K, McIlleron H, Essack S, Padayatchi N. 2017. A Review of Moxifloxacin for the Treatment of Drug-Susceptible Tuberculosis. J Clin Pharmacol 57:1369–1386. https://doi.org/10.1002/jcph.968

78. Guan Y, Liu Y. 2020. Meta-analysis on Effectiveness and Safety of Moxifloxacin in Treatment of Multidrug Resistant Tuberculosis in Adults. Medicine (Abingdon) 99:e20648. https://doi.org/10.1097/MD.0000000000020648

79. Zhang Y, Shi W, Zhang W, Mitchison D. 2014. Mechanisms of Pyrazinamide Action and Resistance. Microbiol Spectr 2:MGM2–0023 https://doi.org/10.1128/microbiolspec.MGM2-0023-2013

80. Laudouze J, Rokitskaya TI, Abolet A, Point V, Firsov AM, Khailova LS, Cavalier J-F, Canaan S, Baulard AR, Antonenko YN, Gouzy A, Santucci P. 2025. Pyrazinamide kills Mycobacterium tuberculosis via pH-driven weak-acid permeation and cytosolic acidification. bioRxiv:2025.09.26.678883. https://doi.org/10.1101/2025.09.26.678883

81. Wehrli W. 1983. Rifampin: mechanisms of action and resistance. Rev Infect Dis 5 Suppl 3:S407–11. https://doi.org/10.1093/clinids/5.supplement_3.s407

82. Telenti A, Imboden P, Marchesi F, Matter L, Schopfer K, Bodmer T, Lowrie D, Colston MJ, Cole S. 1993. Detection of rifampicin-resistance mutations in Mycobacterium tuberculosis. The Lancet 341:647–651. https://doi.org/10.1016/0140-6736(93)90417-F

83. Gan WC, Ng HF, Ngeow YF. 2023. Mechanisms of Linezolid Resistance in Mycobacteria. Pharmaceuticals (Basel) 16:784. https://doi.org/10.3390/ph16060784

84. Dietze R, Hadad DJ, McGee B, Molino LPD, Maciel ELN, Peloquin CA, Johnson DF, Debanne SM, Eisenach K, Boom WH, Palaci M, Johnson JL. 2008. Early and extended early bactericidal activity of linezolid in pulmonary tuberculosis. Am J Respir Crit Care Med 178:1180–1185. https://doi.org/10.1164/rccm.200806-892OC

85. Ivanyi J, Zumla A. 2013. Nonsteroidal antiinflammatory drugs for adjunctive tuberculosis treatment. J Infect Dis 208:185–188. https://doi.org/10.1093/infdis/jit153

86. Cubillos-Angulo JM, Nogueira BMF, Arriaga MB, Barreto-Duarte B, Araújo-Pereira M, Fernandes CD, Vinhaes CL, Villalva-Serra K, Nunes VM, Miguez-Pinto JP, Amaral EP, Andrade BB. 2022. Host-directed therapies in pulmonary tuberculosis: Updates on anti-inflammatory drugs. Front Med 9. https://doi.org/10.3389/fmed.2022.970408

87. Hölscher C, Gräb J, Hölscher A, Müller AL, Schäfer SC, Rybniker J. 2020. Chemical p38 MAP kinase inhibition constrains tissue inflammation and improves antibiotic activity in Mycobacterium tuberculosis-infected mice. Sci Rep 10:13629. https://doi.org/10.1038/s41598-020-70184-x

88. Guerra-De-Blas PDC, Torres-González P, Bobadilla-Del-Valle M, Sada-Ovalle I, Ponce-De-León-Garduño A, Sifuentes-Osornio J. 2018. Potential Effect of Statins on Mycobacterium tuberculosis Infection. J Immunol Res 2018:7617023. https://doi.org/10.1155/2018/7617023

89. Guerra-De-Blas PDC, Bobadilla-Del-Valle M, Sada-Ovalle I, Estrada-García I, Torres-González P, López-Saavedra A, Guzmán-Beltrán S, Ponce-de-León A, Sifuentes-Osornio J. 2019. Simvastatin Enhances the Immune Response Against Mycobacterium tuberculosis. Front Microbiol 10:2097. https://doi.org/10.3389/fmicb.2019.02097

90. Parihar SP, Guler R, Khutlang R, Lang DM, Hurdayal R, Mhlanga MM, Suzuki H, Marais AD, Brombacher F. 2014. Statin Therapy Reduces the Mycobacterium tuberculosis Burden in Human Macrophages and in Mice by Enhancing Autophagy and Phagosome Maturation. J Infect Dis 209:754–763. https://doi.org/10.1093/infdis/jit550

91. Singhal A, Jie L, Kumar P, Hong GS, Leow MK-S, Paleja B, Tsenova L, Kurepina N, Chen J, Zolezzi F, Kreiswirth B, Poidinger M, Chee C, Kaplan G, Wang YT, De Libero G. 2014. Metformin as adjunct antituberculosis therapy. Sci Transl Med 6:263ra159. https://doi.org/10.1126/scitranslmed.3009885

92. Johnson BK, Colvin CJ, Needle DB, Mba Medie F, Champion PAD, Abramovitch RB. 2015. The Carbonic Anhydrase Inhibitor Ethoxzolamide Inhibits the Mycobacterium tuberculosis PhoPR Regulon and Esx-1 Secretion and Attenuates Virulence. Antimicrob Agents Chemother 59:4436–4445. https://doi.org/10.1128/AAC.00719-15

93. Rybniker J, Chen JM, Sala C, Hartkoorn RC, Vocat A, Benjak A, Boy-Röttger S, Zhang M, Székely R, Greff Z, Orfi L, Szabadkai I, Pató J, Kéri G, Cole ST. 2014. Anticytolytic screen identifies inhibitors of mycobacterial virulence protein secretion. Cell Host Microbe 16:538–548. https://doi.org/10.1016/j.chom.2014.09.008
